# Empyema caused by *Actinomyces odontolyticus*: A case report

**DOI:** 10.1097/MD.0000000000037003

**Published:** 2023-02-02

**Authors:** Min Wang, Qianfeng Xiao, Kaige Wang

**Affiliations:** a Department of Pulmonary and Critical Care Medicine, West China Hospital, Sichuan University, Chengdu, China; b Department of Cardiology, West China Hospital, Sichuan University, Chengdu, China.

**Keywords:** *Actinomyces odontolyticus*, biphasic culture, case report, empyema, medical thoracoscopy, metagenome next generation sequencing

## Abstract

**Rationale:**

*Actinomyces odontolyticus* causes a rare, chronic granulomatous infection that is frequently associated with immunocompromised states. *A odontolyticus* can cause infection in multiple organs, but empyema is rare.

**Patient concerns:**

We report a case of empyema caused by *A odontolyticus*. The patient was a 64-year-old man. He was admitted to the hospital with a 5-day history of fever and dyspnea. He had caries and sequelae of cerebral apoplexy.

**Diagnoses:**

Metagenome next generation sequencing of pleural effusion was positive for *A odontolyticus*. Pathogen was identified by biphasic culture of pleural effusion fluid.

**Interventions:**

According to the drug sensitivity test, linezolid 0.6 g twice daily and clindamycin 0.6 g 3 times a day were administered intravenously. Thoracic drainage was initially performed, but the drainage was not sufficient. Medical thoracoscopy was performed to fully drain the pleural effusion.

**Outcomes:**

After anti-infection and medical thoracoscopic therapy, the symptoms of this patient improved.

**Lessons:**

Microbial metagenome sequencing can find pathogens that are difficult to culture by traditional methods. Adequate drainage was the key to the treatment of empyema. Medical thoracoscopy was recommended to remove the pleural effusion and spoilage when thoracic drainage is difficult. The common clinical features of *A odontolyticus* include a mass or swelling, abdominal disease, dental disease, and subcutaneous abscesses. Microbial metagenome sequencing can find pathogens that are difficult to culture by traditional methods. Adequate drainage was the key to the treatment of empyema. Medical thoracoscopy was recommended to remove the pleural effusion and spoilage when thoracic drainage is difficult.

## 1. Introduction

Actinomycosis is a chronic, suppurative, granulomatous disease caused by actinomycetes. Lesions often occur on the face and neck, as well as in the thorax and abdomen. Actinomycosis easily spreads to surrounding tissues and forms fistulas, accompanied by sulfur-like granular pus effusion. *Actinomyces odontolyticus* was first isolated from the oral cavity by Batty in 1958^[1]^ and can cause lung, abdominal cavity, blood, brain, pericardial cavity and bone marrow infections.^[2,3]^ However, there are few reports about thoracic infections caused by *A odontolyticus*.^[4–6]^ We report a case of empyema caused by *A odontolyticus* and analyze the clinical characteristics and treatment strategies to improve the clinical diagnosis and treatment level of this bacterium. We present the following case in accordance with the CARE reporting checklist.

## 2. Case presentation

A 64-year-old man was admitted to the hospital with a 5-day history of fever and dyspnea. His temperature was as high as 38.7ºC without chills. He was treated with piperacillin/tazobactam and levofloxacin in a local hospital, without any improvement, and was then transferred to our department. Ten years ago, he underwent craniotomy because of cerebral hemorrhage and then experienced weakness in the right limbs. In addition, he had dental caries.

**Figure 1.**
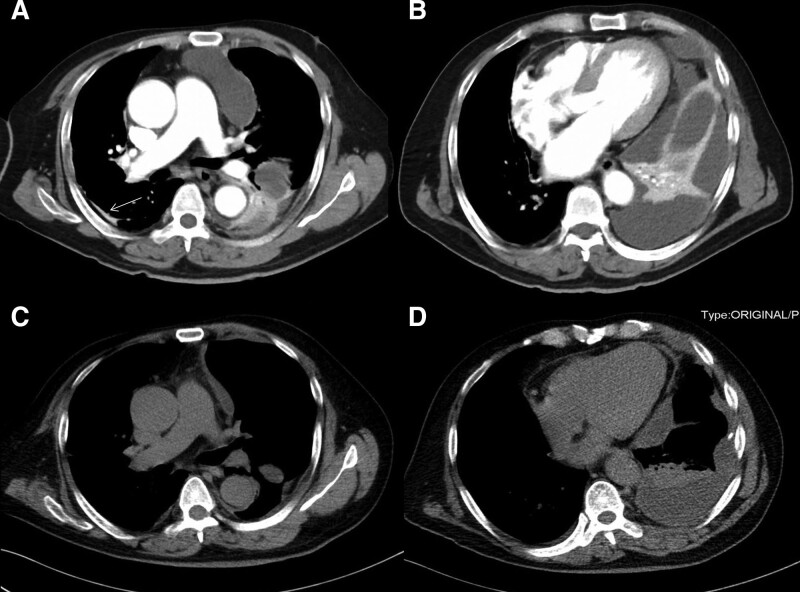
Image of Thoracic CT. (A and B) Thoracic CT scan on admission. The image showed a massive pleural effusion on the left side, which was wrapped locally, and local atelectasis in the right lung. (C and D) Thoracic CT scan on d 10 of hospitalization. The image showed that the left pleural effusion became smaller, with local encapsulation. CT = computed tomography.

**Figure 2.**
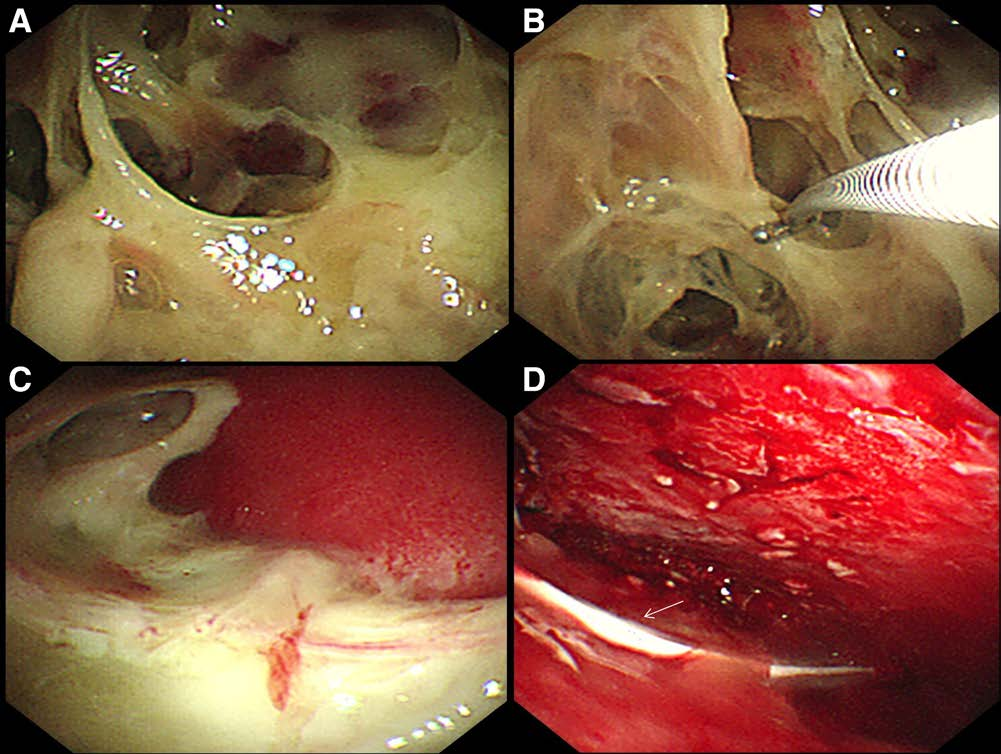
Image of medical thoracoscopy. (A) The image showed fibrous separation and necrotic tissues in the pleural cavity. (B) The image showed that necrotic tissues were removed through medical thoracoscopy.

**Figure 3.**
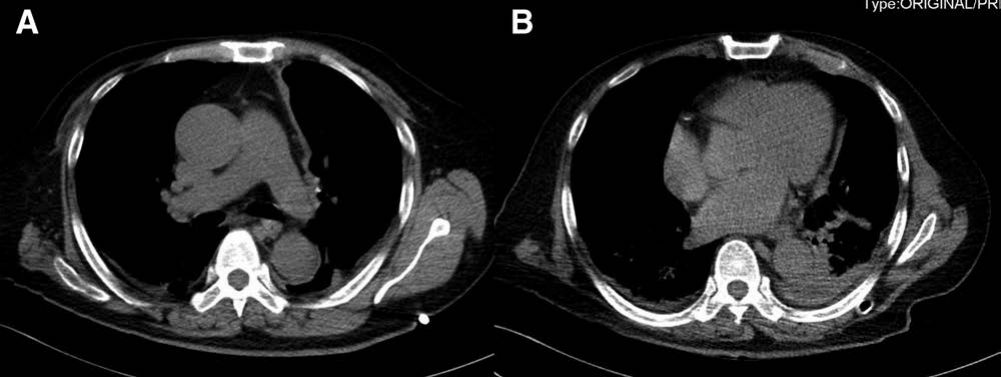
Image of Thoracic CT. (A and B) Thoracic CT scan on d 18 of hospitalization. The image showed that the left pleural effusion was obviously absorbed. CT = computed tomography.

On admission, his breathing rate was 30/min, percussion revealed flatness in the lower zone of left lung, and auscultation revealed low breath sounds in the mid zone and lower zone of left lung. The water swallow test was normal (Level 6, Level 1: Dysphagia and inability to swallow under any condition; Level 2 to 5: There are different degrees of swallowing dysfunction. Level 6: Normal swallowing). Arterial blood gas analysis showed that the partial pressure of oxygen was 50 mm Hg without oxygen. The peripheral blood white cell count was increased to 14 × 10^9^/L (reference, 4.0–10 × 10^9^/L). Both C-reactive protein and procalcitonin were increased, with values of 190 mg/L (reference, 0–5 mg/L) and 1.29 pg/L (reference, 0–0.05 pg/L), respectively. Thoracic computed tomography (CT) showed a massive pleural effusion on the left side, which was wrapped locally, and local atelectasis in the right lung (Fig. 1A and B). Approximately 800 mL of yellow, purulent pleural effusion fluid was drained by ultrasound-guided left thoracic catheterization (diameter 14F). Purulent cells from the pleural effusion were positive under the microscope. Biochemical examination showed the total protein of 42.1 g/L, glucose of <0.1 mmol/L, lactate dehydrogenase of 19,070 IU/L, and adenosine deaminase of 185 IU/L. Microbiological culture of pleural effusion fluid on chocolate agar plates was negative 3 times after 7 days. Metagenome next generation sequencing (mNGS) of pleural effusion by a commercial test (Explify Respiratory) was positive for *A odontolyticus*. Pleural effusion fluid was injected into a biphasic blood culture bottle, *A odontolyticus* grew 24 hours later in an anaerobic bottle. Drug sensitivity tests showed that the culture was resistant to penicillin G and meropenem, and sensitive to clindamycin, and linezolid. Consistent culture results were obtained 1 day later. The patient was diagnosed with left empyema, right local atelectasis, respiratory failure. Therefore, linezolid 0.6 g twice daily and clindamycin 0.6 g 3 times a day were administered intravenously. By the 10th day of hospitalization, the dyspnea significantly improved, but he still had fever, and the highest temperature was 38ºC. Thoracic CT (Fig. 1C and D) showed that the left pleural effusion became smaller, with local encapsulation. Thoracic ultrasound showed localized pleural effusion with severe separation. Thoracic catheterization was very difficult. Then we chose the 7th intercostal space of the left posterior axillary line as the puncture point, 1 centimeter incision was made along the intercostal space, and then the trocar was inserted. Medical thoracoscopic treatment was performed, and fibrous separation and necrotic tissues were found in the pleural cavity (Fig. 2A and B). We extracted 300 mL pus, removed necrotic tissues and then rinsed the thoracic cavity with saline (Fig. 2C and D). After that, no fever occurred in the patient. Pleural pathologic biopsy showed fibrous tissue hyperplasia and inflammatory granulation tissue formation, with inflammatory necrotic exudate, while PAS staining, acid-fast staining, and hexamine silver staining were negative. On the 18rd day of hospitalization, Thoracic CT (Fig. 3A and B) showed that the left pleural effusion was obviously absorbed, and the patient was discharged. After discharge, He returned to normal after 7 days of oral linezolid. Diagnosis and treatment of the patient and changes in his condition (Table 1). All procedures performed in studies involving human participants were in accordance with the ethical standards of the institutional and/or national research committee(s) and with the Helsinki Declaration (as revised in 2013). Written informed consent was obtained from the patient.

**Table 1:** Flow of diagnosis and treatments.

Indexes	Normal range	Hospitalization				Discharged from hospital 7 d after discharge
		D 1	D 4	D 10	D 18 (discharge)	
Temperature (ºC)		38.9	38.5	38.0	36.4	36.5
Whole blood cell count (×10^9^/L)	3.5–9.5	14.0	15.2	13.6	8.2	6.8
Neutrophil (%)	40–70	1.29	1.36	0.55	˂0.02	
PCT (ng/mL)	0–0.05	1.29		0.55	˂0.02	
Image		Figure 1A and B		Figure 1C and D	Figure 3A and B	
Local treatment		Thoracic drainage		Medical thoracoscopy		
Drugs		Piperacillin tazobactam 4.5 g q8h, Levofloxacin 0.5 g QD	Linezolid 0.6 g tid, Clindamycin 0.6 g tid			Linezolid 0.6 g tid PO

## 3. Discussion

In this study, we described a case of empyema caused by *A odontolyticus* infection. mNGS and anaerobic culture of pleural effusion fluid confirmed the pathogen. Hooi reported a case of empyema with mixed infection of *A odontolyticus* and anaerobes, which was cured by thoracic drainage and penicillin injected intravenous.^[4]^ For encapsulated empyema caused by *A odontolyticus*, the therapeutic effect of medical thoracoscopy has not been reported.^[7]^

*A odontolyticus* is a V- and Y-shaped gram-positive bacillus that is negative for acid-fast staining, immotile and nonsporulating. There are sometimes short or medium-sized rods similar to those of the diphtheria bacillus, which can grow in the presence or absence of CO_2_, with the best growth in an anaerobic environment.^[1]^ Most patients with empyema have anaerobic infections, and therefore, biphasic culture of pleural effusion fluid should be conducted. Microbial metagenome sequencing can find pathogens that are difficult to culture by traditional methods. For patients with infectious pleural effusion, if the etiological diagnosis cannot be confirmed by routine laboratory examination, the mNGS of pleural effusion should be performed to assist in the diagnosis. Poor oral hygiene, diabetes and alcoholism are risk factors for pulmonary actinomycosis.^[8]^ Immunodeficient patients are more likely to suffer from pulmonary actinomycosis, but it can also be observed in people with normal immunity. Most patients infected with *A odontolyticus* have other pathogen infections, with *Streptococcus* spp. accounting for 50%.^[9]^ Caries may be the potential cause of infection in this patient.

The common clinical features of actinomycosis include a mass or swelling, lung disease, abdominal disease, dental disease and intracranial infection. Empyema caused by *A odontolyticus* can cause subcutaneous abscesses and sinuses by penetrating the thoracic wall^[4,10]^ and even respiratory failure in severe patients.^[11]^ Ashley Gray reported a case of a child with endobronchial *A odontolyticus* infection presenting with cystic bronchiectasis on thoracic CT.^[12]^ The key to successful treatment of empyema is the combination of appropriate anti-infection drug therapy and pus drainage. Medical thoracoscopy can reduce pleural separation and adhesion of empyema and help accurately insert the thoracic drainage tube.^[7]^

## 4. Conclusions

*A odontolyticus* is a rare pathogen in patients with empyema, mNGS and biphasic culture of pleural effusion fluid is helpful to identify the pathogen. The treatment of empyema, especially that with a poor response, not only requires identification of the type of the pathogen and anti-infection agents according to the drug sensitivity of the pathogen but also requires clearance and drainage of thoracic necrotic tissues. Furthermore, medical thoracoscopic treatment is recommended when thoracic drainage is difficult.

## Author contributions

**Investigation:** Qianfeng Xiao.

**Methodology:** Qianfeng Xiao.

**Project administration:** Kaige Wang.

**Supervision:** Kaige Wang, Qianfeng Xiao.

**Writing – original draft:** Min Wang.

**Writing – review & editing:** Kaige Wang.
